# Biomaterials derived from hard palate mucosa for tissue engineering and regenerative medicine

**DOI:** 10.1016/j.mtbio.2023.100734

**Published:** 2023-08-05

**Authors:** Lingfei Ren, Zhiwei Jiang, Hui Zhang, Yani Chen, Danji Zhu, Jin He, Yunxuan Chen, Ying Wang, Guoli Yang

**Affiliations:** Stomatology Hospital, School of Stomatology, Zhejiang University School of Medicine, Zhejiang Provincial Clinical Research Center for Oral Diseases, Key Laboratory of Oral Biomedical Research of Zhejiang Province, Cancer Center of Zhejiang University, Engineering Research Center of Oral Biomaterials and Devices of Zhejiang Province, Hangzhou, 310000, China

**Keywords:** Autologous biomaterials, Hard palate mucosa, Tissue engineering, Regenerative medicine

## Abstract

Autologous materials have superior biosafety and are widely used in clinical practice. Due to its excellent trauma-healing ability, the hard palate mucosa (HPM) has become a hot spot for autologous donor area research. Multiple studies have conducted an in-depth analysis of the healing ability of the HPM at the cellular and molecular levels. In addition, the HPM has good maneuverability as a donor area for soft tissue grafts, and researchers have isolated various specific mesenchymal stem cells (MSCs) from HPM. Free soft tissue grafts obtained from the HPM have been widely used in the clinic and have played an essential role in dentistry, eyelid reconstruction, and the repair of other specific soft tissue defects. This article reviews the advantages of HPM as a donor area and its related mechanisms, classes of HPM-derived biomaterials, the current status of clinical applications, challenges, and future development directions.

## Highlights

• The hard palate mucosa (HPM), holds promise as an ideal donor area for autologous biomaterials.
• The advantages of HPM as a donor area and its related mechanisms are critically reviewed.
• The categories and the current clinical applications of HPM-derived biomaterials are particularly presented.
• The challenges and future directions of HPM-derived biomaterials are discussed.

## Graphical abstract

**Figure:**
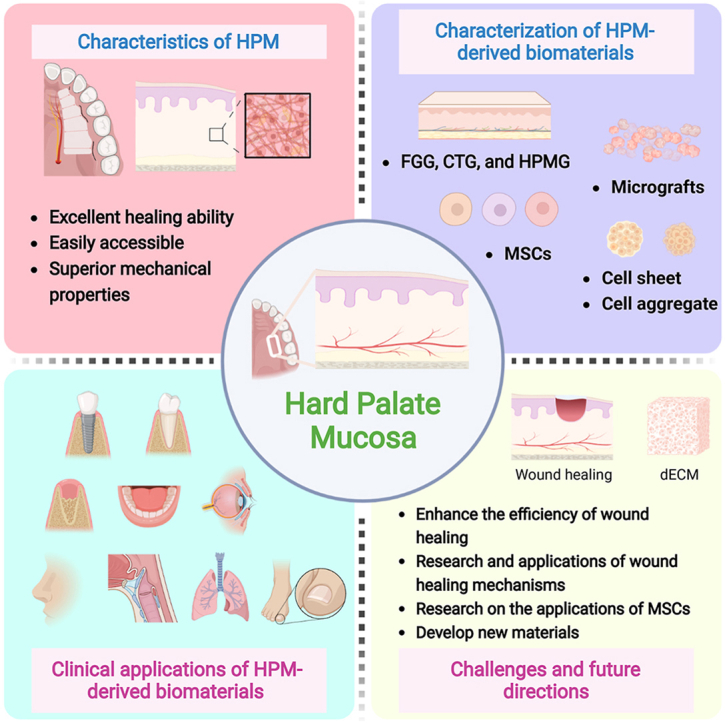

## Abbreviations

HPM: hard palate mucosa
FGG: free gingival graft
SCTG: subepithelial connective tissue graft
HPMG: hard palate mucosa graft
MSC: mesenchymal stem cell
KTW: keratinized tissue width
miR: microRNA
VEGF: vascular endothelial growth factor
TGF-β1: transforming growth factor-b1
IL-6: interleukin-6
HIF-1α: hypoxia-inducible factor 1α
ECM: extracellular matrix
HMGB1: high mobility group box 1
RAGE: receptor for advanced glycation end product
S1P: sphingosine 1-phosphate
S1P1: sphingosine 1-phosphate 1
SOX2: sex-determining region Y-box 2
PITX1: paired-like homeodomain 1
GPA: greater palatal artery
CBCT: cone beam computed tomography
AMG: autologous micrograft
PMSC: palate-derived mesenchymal stem cell
MPE: medicinal plant extracts
IL-10: interleukin-10
TNF-α: tumor necrosis factor-α
dECM: decellularized extracellular matrix
3D: three-dimensional

## 1 Introduction

Autologous materials are significant in tissue engineering and regenerative medicine [1]. Autologous materials have the best safety profile, but the donor area often influences autologous biomaterials. The ideal donor area demands adequate space for manipulation, acceptable post-operative complications, and the ability to repeat retrieval [2]. The hard palate mucosa (HPM) is a hard, immovable tissue with dense collagen fibers and a rich capillary network [3]. Its grafts have excellent healing ability and superior mechanical properties; the anatomy and location of the hard palate provide sufficient space for retrieval and good clinical maneuverability.

HPM-derived biomaterials were put into clinical use more than 50 years ago, and free gingival graft (FGG), obtained by surgical excision directly from the HPM, were first used for increasing keratinized tissue [4]. The subepithelial connective tissue graft (SCTG) proposed obtained better aesthetic results and was widely used in oral surgeries [5]. Later, some surgeons applied the hard palate mucosa graft (HPMG) in the eyelid and other special soft tissue reconstruction [6]. Many researchers have extracted new mesenchymal stem cells (MSCs) from the HPM for bone regeneration, periodontal tissue repair, and tissue homeostasis studies in the last decade [[7], [8], [9]]. HPM-derived biomaterials are not only polymorphic but multifunctional.

To date, the clinical applications of HPM-derived soft tissue grafts have been quite extensive, mainly including keratinized tissue width (KTW) augmentation [10,11], soft tissue augmentation [10,12], root coverage [13,14], alveolar ridge preservation [15,16], eyelid reconstruction [17,18], contracted anophthalmic socket reconstruction [19], nail bed reconstruction [20,21], lip defect reconstruction [22], nasal vestibular stenosis reconstruction [23], posttraumatic nasal deformity correction [24], hypopharyngeal defect reconstruction [25], and tracheal reconstruction [26]. Notably, except for soft tissue grafts, most HPM-derived biomaterials are in the experimental stage and have a long way to go before they can be used clinically.

In addition, wounds in the donor site can narrow the application range of HPM-derived biomaterials. Therefore, researchers are dedicated to analyzing the trauma-healing mechanism of HPM to improve its healing ability further and advance the clinical applications of HPM-derived biomaterials [2].

This review summarizes the advantages of HPM, the related mechanisms as a donor area, and the forms of HPM-derived biomaterials. In addition, the current status of clinical applications is presented. Furthermore, we discuss the challenges and progress of HPM-derived biomaterials to provide an outlook on future directions.

## 2 Characteristics of HPM and related mechanisms

**Fig. 1:**
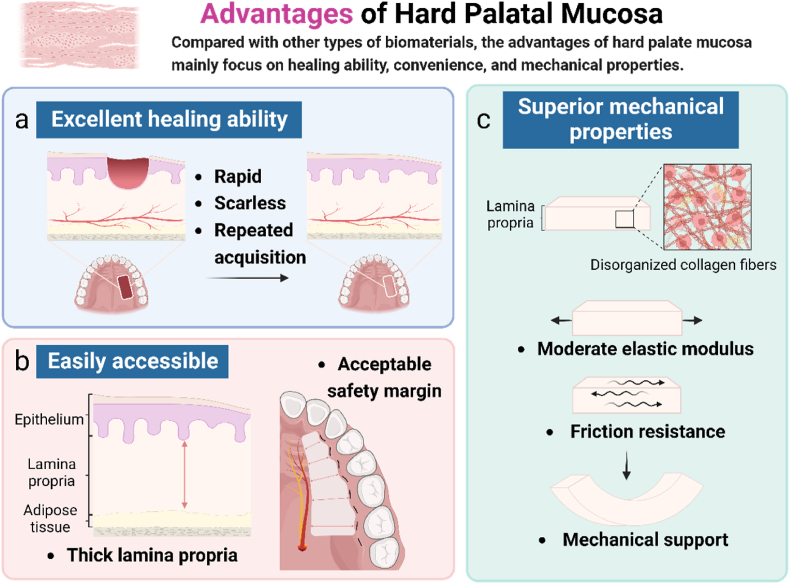
The advantages of HPM. a) HPM has excellent healing ability, including rapid healing, scarless, and repeated acquisition. b) The thicker lamina propria, the deformed nerve and blood vessels, and other anatomical features make it easy to obtain HPM-derived biomaterials. c) The lamina propria of the HPM is composed of dense, thick, and disorderly collagen fibers, so it has moderate elastic modulus, good friction resistance, and mechanical support ability. Created with BioRender.com.

Conventional autograft acquisition is often accompanied by donor zone trauma, patient pain, and certain post-operative complications, which can cause irreversible damage to the donor zone in severe cases [27,28]. Therefore, researchers have been searching for a better autograft donor area. The HPM, with its unique anatomy and composition, is characterized by excellent wound healing ability, low clinical difficulty, and large donor area, and its tissue grafts have particular physical properties. Researchers have conducted various studies to gain insights into the characteristics of HPM and related mechanisms (Fig. 1).

### 2.1 Excellent healing ability

**Fig. 2:**
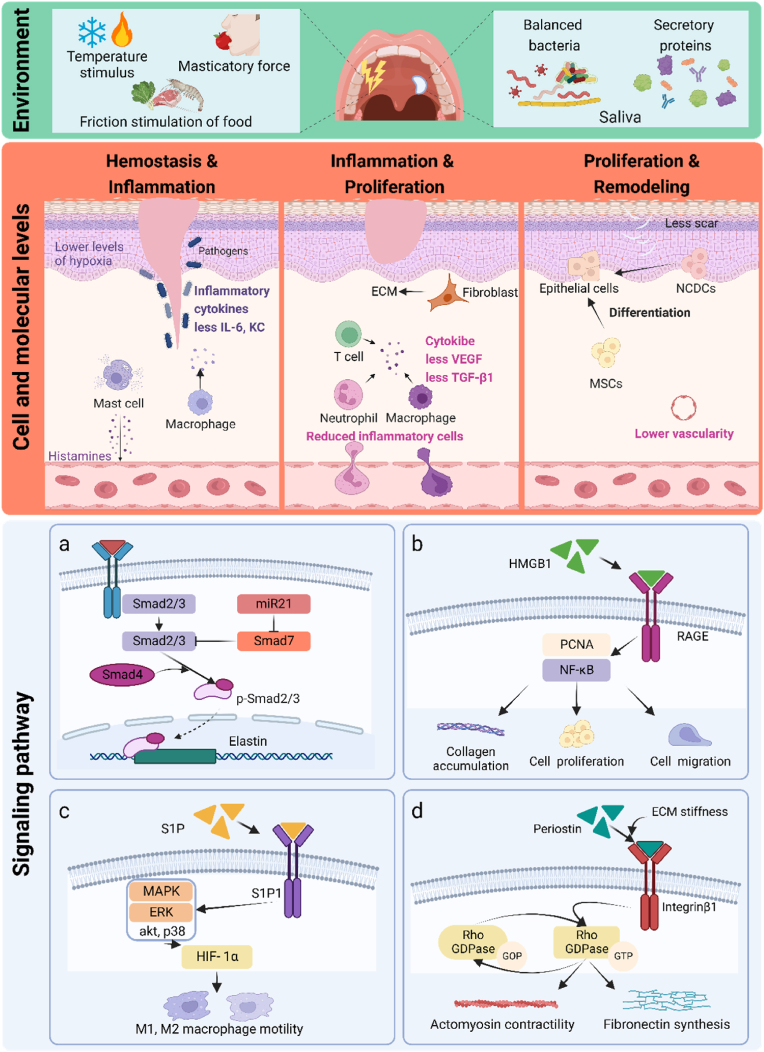
Factors related to the superior healing ability of the HPM compared to skin wounds. At the level of the oral microenvironment, the HPM wounds are affected by various stimuli in the oral cavity, including food friction, temperature, masticatory pressure, oral microorganisms, and saliva. At the cellular and molecular levels, MSCs in the HPM can promote wound healing; inflammation-associated cells at the wound site are less, with the low secretion of inflammatory factors (IL-6 and KT) and cytokines (TGF-β1 and VEGF). Some studies focused on signaling pathways. a) The miR-21 promotes wound healing through the Smad7-Smad2/3-elastin pathway. b) The HMGB1/RAGE axis is essential in palatal wound healing. c) HIF-1α controls the conversion of M1 and M2 macrophage phenotypes through S1P/S1P1 signaling. d) Periostin modulates myofibroblast differentiation and contraction via integrinβ1/RhoA pathway and fibronectin synthesis in an ECM stiffness-dependent manner in HPM wound healing. Created with BioRender.com.

Wound healing is a complex process divided into three overlapping phases: inflammation, proliferation, and remodeling [29]. These phases involve a complex network of cells, molecules, cytokines, and microRNAs (miRs) [30]. The HPM, the part of oral mucosa, has long been considered one of the optimal wound resolutions characterized by rapid and scarless wound healing [31]. As the body's main external barrier, the skin is a frequent site of tissue damage [30]. Improving skin wound healing resolution is a major medical and social priority. Therefore, many researchers compare the microenvironmental, cellular, and molecular differences in the healing process of hard palate mucosa and skin wounds in order to elucidate the mechanisms underlying the excellent healing ability of hard palate mucosa and to explore the breakthroughs to enhance the healing ability of skin wounds [31,32] (Fig. 2).

There are billions of bacteria, fungi, and viruses in the oral cavity (oral microbiota), and oral microorganisms are always in dynamic ecological balance with the oral and human body states, which has major effects on physical health [33]. A recent study suggested that the balance between pathogenic and probiotic bacteria in the oral cavity promotes the migration, osteogenic differentiation, and cell proliferation of MSCs. This study further pointed out that reuterin, the effective ingredient in *L. reuteri*, maintained the balance of pathogenic bacteria and probiotics by neutralizing lipopolysaccharide in *P. gingivalis*, thus inhibiting inflammation and promoting wound healing [34]. Furthermore, it has been proposed that stimuli such as hot and cold stimuli, chewing food, and biting friction inside the oral mucosa might also affect wound healing [31]. In addition, the HPM is completely infiltrated with saliva, and research on saliva and oral wounds has observed that secreted proteins in saliva positively impact oral mucosal wound healing [34]. One more intense research has highlighted that vascular endothelial growth factor (VEGF) in saliva plays a vital role in wound closure and vascular density [35].

Research on stem cells has revealed that neural crest-derived cells in the form of stem cells are present in HPM and have the potential to differentiate into multiple cell types during the wound-healing process, thereby promoting wound healing [36].

At the molecular level, studies have shed more light on the mechanisms underlying the healing ability of the HPM. Firstly, studies at the phenomenological level found that oral wounds have significantly lower levels of transforming growth factor-b1 (TGF-β1), interleukin-6 (IL-6), and KC (a murine homolog of human interleukin-8) than skin wounds [37,38]. Oral wounds showed significantly fewer vascular changes and lower levels of VEGF, and specific differences in angiogenesis suggested that skin and oral mucosal wounds may experience different levels of hypoxia and hypoxia-inducible factor 1α (HIF-1α). Then, using a mouse model of trauma, higher levels of hypoxia were found in skin wounds than in mucosal wounds, along with differences in HIF-1α expression [39].

Other researchers have focused on signaling pathways. R. MA et al. found that miR-21 promotes oral wound healing by inhibiting autophagy and increasing extracellular matrix (ECM) [40]. Xiaoyan Li's team also investigated miR-21 and found that miR-21 promotes wound healing through the Smad7-Smad2/3-elastin pathway (Fig. 2a) [41]. High mobility group box 1 (HMGB1) is closely related to the healing process after tissue injury. Salunya et al. found that inhibition of receptors for advanced glycation end product (RAGE) prevents rHMGB1-induced cell migration and proliferating cell nuclear antigen expression (Fig. 2b) [42]. A further study targeting HIF-1α revealed that HIF-1α controls the conversion of M1 and M2 macrophage phenotypes through sphingosine 1-phosphate/sphingosine 1-phosphate 1 (S1P/S1P1) signaling and controls macrophage motility, thereby affecting wound healing in the HPM (Fig. 2c) [43]. Georgia et al. concluded that Periostin modulates myofibroblast differentiation and contraction via integrinβ1/RhoA pathway and fibronectin synthesis in an ECM stiffness-dependent manner in HPM wound healing (Fig. 2d) [44].

Some investigators looked at the overall gene expression level to analyze differences in wound healing between the oral and skin. Early in 2010, a research team used microarray analysis in a mouse model to determine differences in gene expression in skin and oral mucosal wound healing, suggesting that such differences in gene levels may cause differential responses to injury [45].

Two-cell tight junctions are multiprotein complexes that maintain barrier function and windowing in epithelial tissues. Trevor R. Leonardo et al. reanalyzed mouse skin and palatal epithelia microarray datasets as well as skin and tongue wound healing microarray datasets. They found that oral and skin epithelia express different tight junction genes at baseline and during wound healing responses [46].

Ramiro Iglesias Bartolome et al. then analyzed human oral and skin sampling and found that wound-activated transcriptional networks are present in the basal state of the oral mucosa, priming the epithelium for wound repair. Furthermore, this study also pointed out that differentially expressed sex-determining region Y-box 2 (SOX2) and paired-like homeodomain 1 (PITX1) confer a unique identity to oral keratinocytes. Moreover, SOX2 and PITX1 transcriptional functions can potentially reprogram skin keratinocytes to increase cell migration and improve wound resolution *in vivo* [31].

### 2.2 Easily accessible

The HPM is located anterior to the maxilla. In most patients with standard mouth opening, the surgeon can obtain the appropriate graft from the region of HPM through outpatient surgery with the patient under local anesthesia [32]. The safe operating range of HPM is closely related to the location of the greater palatal foramen and the alignment of the greater palatal artery (GPA) [47]. In a systematic review by Tavelli et al., 91.87% of the greater palatine foramina were located between the palatal sides of the second and third molars or more distally. As it traverses the palate anteriorly, the distance from the GPA to the maxillary teeth gradually changes; the least distance from the GPA to the teeth was found in the canine area (9.9 ± 2.9 mm), whereas the greatest distance was in the second molar region (13.9 ± 1 mm) [48].

Many authors have comprehensively analyzed the thickness of the HPM donor area through clinical bone probing [49,50], cone beam computed tomography (CBCT) [47,51], ultrasonography [52], and cadaveric specimens [53]. Various factors influence the thickness of the HPM, including age, gender, race, smoking status, dentition, orthodontic treatment, systemic disease, medications, immunosuppression, and individual variation [47,51,53]. Therefore, it has been proposed that combined data from CBCT scans and intraoral surface scans allow a three-dimensional volumetric analysis of the HPM, which can be used to determine the thickness of the donor area by a noninvasive method during the planning phase prior to surgical intervention [54].

### 2.3 Superior mechanical properties

These superior mechanical properties are compared to other soft tissue grafts, including buccal mucous membrane graft, full-thickness skin graft, vascularized pedicle flaps, dermis fat graft, and dermal fillers [19]. In addition, this comparison regarding mechanical properties is specific to the form and function that needs to be restored in the receiving area [18,55].

The oral soft tissues are a complex biological system with extracellular matrix components that respond differently to physiological stress. These tissues are subjected to various mechanical forces, including salivary flow, hydrodynamic forces generated during mastication and speech, compression, tension, friction, and shear [55,56]. The oral mucosa consists of different fibers located at different locations in the oral cavity, with different structural compositions, subjected to different ranges of masticatory loads, and with different physical properties such as stiffness, elastic modulus, and tensile properties [55]. As a heterogeneous material, factors affecting the physical properties of the mucosa come from the solid matrix structure and the fluid composition [57].

A cadaveric study by J.J.E. et al. found that the hard palate soft tissue graft had a mean elastic modulus of 18.1 ± 4.5 MPa. Furthermore, tissue showed dense fibers and a randomly oriented reticular organization; the palatal mucosa had a high density of collagen fibers (thicker fibers) but poor directionality; and the collagen content of HPM was intermediate between that of the keratinized gingiva and the buccal mucosa. In addition, the study examined the palatal mucosa by scanning electron microscopy and found a relatively random orientation of collagen arrangement in its lamina propria, which might explain its unique physical properties [56].

## 3 Characterization of HPM-derived biomaterials

**Fig. 3:**
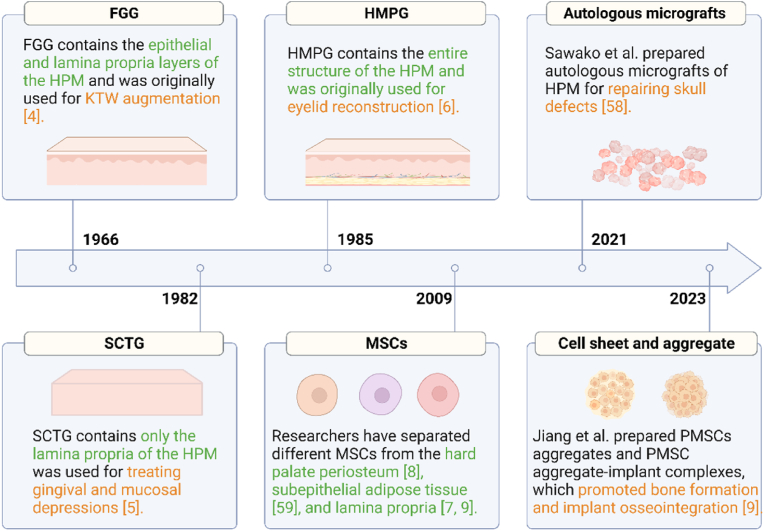
Timeline | Key discoveries and advances in the development of HPM-derived biomaterials [[4], [5], [6], [7], [8], [9],^58^,^59^]. Created with BioRender.com.

After discovering the above advantages of the HPM, researchers have developed a variety of biomaterials from it, including FGG, SCTG, HPMG, and autologous micrografts (AMG), and HPM-derived MSCs (Fig. 3).

### 3.1 FGG, SCTG and HPMG

Soft tissue grafts with overlying epithelium collected from HPM are defined as FGG [4]. FGG is the first palatal mucosal biomaterial to be clinically applied and was obtained by surgical excision directly from the HPM, containing the epithelial and lamina propria layers of the HPM [60]. The procedure follows: 2 horizontal and vertical incisions of approximately graft size are made with the blade perpendicular to the mucosal surface 2 mm below the gingival margin of the hard palate. The horizontal and vertical incisions need to be extended slightly to form a crossover to the required thickness of the graft. And then, the blade is turned parallel to the mucosal surface, and the palatal tissue is cut from the proximal to the distal center of the horizontal incision on the coronal side, moving toward the root to remove the FGG [61].

Most FGGs are clinically obtained with the periosteum preserved on the donor area. Studies have found that periosteum loss can interfere with wound healing and lead to increased pain in the operative area, and in still-developing jaws, periosteum loss can lead to developmental deformities [62].

Keratinized gingiva reconstruction is the most prominent application for FGG, including KTW augmentation [4], alveolar ridge preservation [63], root coverage [13], and alveolar ridge preservation [64].

SCTG has a relatively homogeneous tissue structure, preserving only the lamina propria of the HPM [5]. The further development from FGG to SCTG is an essential milestone in periodontal soft tissue management and implies the beginning of the transition from classical mucosal surgery to periodontal plastic surgery [61].

The most common method of obtaining SCTG clinically is to remove the epithelial layer with a blade parallel to the FGG, while scraping away the underlying fat and glandular tissue [5]. Alternatively, surgical techniques such as trap-door [65] and single incisions [66] can also be used to preserve the epithelium and obtain SCTG directly from HPM, thus achieving initial healing of the palatal wound.

To date, SCTG has become the technique of choice for treating gingival and mucosal depressions in dental and implant sites [12], increasing soft tissue thickness [10], masking discolored roots or visible implant components [13], and interdental papilla reconstruction [14].

HPMG is obtained similarly to FGG, with a deeper incision, and will be removed along with the periosteum or fat layer, depending on clinical needs [20,67]. HPMG is generally applied for eyelid, nail bed, and other special soft tissue reconstruction. In particular, HPMG for eyelid reconstruction requires additional periosteum for better support [68].

### 3.2 Autologous micrografts

The AMG technique is an emerging therapy for skin injuries that can improve wound healing, minimize scar formation, and represents an affordable alternative to traditional skin grafting [69]. AMGs are free tissue suspensions from tissue grafts containing stem/progenitor cells, growth factors, and ECM components [70].

A suitable autologous soft tissue graft is integral to AMG treatment [71]. HPM is an ideal donor for AMGs because of its ease of operation and rapid wound healing compared to other autologous soft tissue donor areas. Sawako et al. obtained the palatal mucosa of Sprague-Dawley rats, removed its epithelial tissue, and used a tissue dissociation apparatus to prepare a dissociated soft tissue suspension containing 1 mL of 10% sucrose solution for the repair of autologous cranial defects, and found that it promoted bone regeneration *in vivo* [58]*.*

### 3.3 MSCs

Adult MSCs are typically found in specialized stem cell ecotopes that provide the microenvironment, growth factors, intercellular contacts, and external signals needed for maintaining stem cell stemness and differentiation capacity [72]. Available sources of adult MSCs include bone marrow, adipose tissue, dental pulp, peripheral blood, transmural blood, and muscle [73]. Autologous MSCs do not undergo immune rejection, have good cell compatibility, and are free of ethical barriers, making them ideal cells for tissue engineering and regenerative medicine applications [74].

In the last 15 years, many researchers have suggested that the hard palate mucosa may be an ideal source of autologous MSCs because of its wound healing ability and easy tissue accessibility [8,9,59,75,76].

The main methods for isolating MSCs from the hard palate mucosa are the enzyme-digested culture method [7] and the outgrowth method [9]. Daruis et al. first isolated neural crest-associated stem cells from the HPM of adult rats, hypothesizing that these cells might be able to differentiate into functional neurons and glial cells [7]. Since then, other researchers have separated different MSCs from the periosteum [8], subepithelial adipose tissue [59], and lamina propria [9].

The characteristics of MSCs isolated from different locations of the hard palate mucosa varied. Flow cytometry data showed positive rates for periosteal-derived MSCs with CD73, CD105, CD90, SSEA-4, and CD34 of 99%, 95%, 48%, 56%, and 0%, respectively [8]. And subepithelial adipose tissue-derived MSCs displayed positive staining for the mesenchymal markers CD29, CD73, CD105, CD 49e, and CD44, but did not express hematopoietic markers CD34/45 [59]. For lamina propria-derived MSCs, results of flow cytometry indicated that the MSCs were positive for CD90, CD44, and CD29 and negative for CD34, CD45, and CD146 [9]. After culture in osteogenic, adipogenic, and chondrogenic medium, all of the different sources of MSCs were positive for alizarin red staining, oil red O staining, and alcian blue staining [8,9,59,75,76]. In addition, some researchers have also cultured MSCs in a neuronal differentiation medium, and the cells gradually transformed into neuronal morphology and expressed various neuronal markers [76].

Although HPM-derived MSCs have not been studied for clinical applications, some studies have explored their application in animal models. Jiang et al. isolated a type of MSCs, named hard palate-derived mesenchymal stem cells (PMSCs), from the lamina propria of rat HPM and prepared cell aggregates by serum-free culture, and formed cell polymers through a light-controlled method. Research showed that PMSC aggregates and PMSC aggregate-implant complexes significantly promoted bone formation in a tibial defect model and implant osseointegration in a tibial implant model [9].

## 4 Clinical applications of HPM-derived biomaterials

**Fig. 4:**
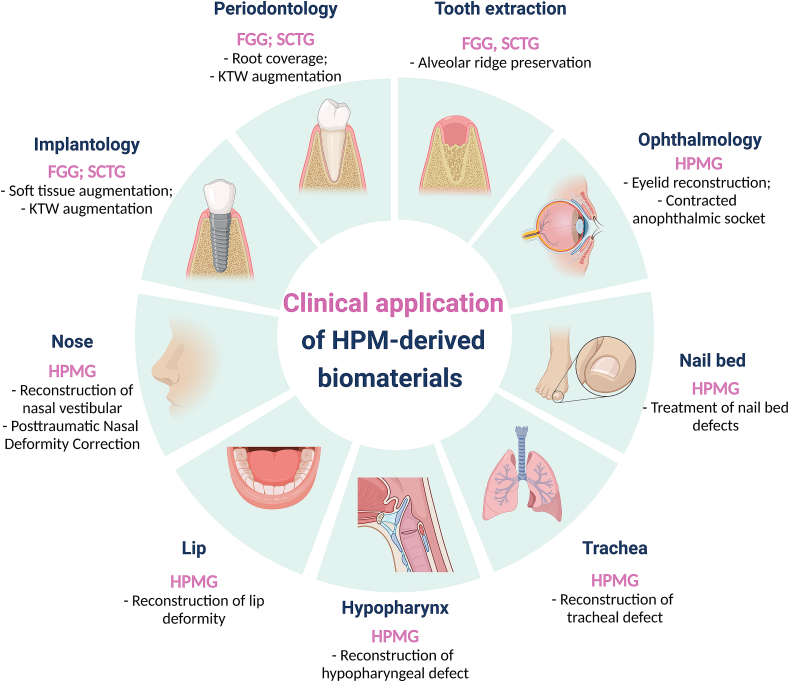
HPM-derived biomaterials, including FGG, SCTG, and HPMG, performs essential and diverse functions in clinical applications of dental implantology, periodontal medicine, ophthalmology, nasal plastic surgery, nail bed, lips, hypopharynx, and tracheal reconstruction. Created with BioRender.com.

Among the HPM-derived biomaterials, FGG, CTG, and HPMG have been widely used in clinical practice, mainly in ophthalmology, dentistry, and other special soft tissue reconstruction fields (Fig. 4). Specific applications include KTW augmentation [10,11], soft tissue augmentation [10,12], root coverage [13,14], alveolar ridge preservation [15,16], eyelid reconstruction [17,18], contracted anophthalmic socket reconstruction [19], nail bed reconstruction [20,21], lip defect reconstruction [22], nasal vestibular stenosis reconstruction [23], posttraumatic nasal deformity correction [24], hypopharyngeal defect reconstruction [25], and tracheal reconstruction [26] (Table 1).

**Table 1:** Clinical applications of HPM-derived soft tissue grafts.

Clinical applications	Biomaterials	Year of Publication	Study design	Treatment position	Number of cases	Follow-up time/month	Related conclusions
KTW augmentation	FGG	2017	Meta	Natural teeth	–	–	FGG achieves a larger and more predictable increase in KTW with poor aesthetics [10].
	FGG	2021	Meta	Peri-implant	–	–	An apically positioned flap in combination with FGG was the most effective technique for increasing KTW [11].
Soft tissue augmentation	SCTG	2019	Meta	Peri-implant	–	–	SCTG is more effective than xenogeneic collagen matrix in improving peri-implant soft tissue thickness [12].
	SCTG	2017	Meta	Natural teeth	–	–	Bilaminar approach involving SCTG obtained the highest amount of mucosal thickness gain [10].
	SCTG	2009	Meta	Natural teeth	–	–	Using SCTGs resulted in statistically significantly more soft tissue volume gain than FGGs [77].
Root coverage	FGG, SCTG	2002	Systematic review	Natural teeth	–	–	FGG achieves effective root coverage but is inferior to SCTG [78].
	FGG	2020	Narrative review	Natural teeth	–	–	Several features were suggested as risk factors for the outcomes of FGG: poor color match with the surrounding tissue, significant shrinkage, poor adaptation to the recipient bed, and failure to stabilize the graft [13].
	SCTG	2020	Meta	Natural teeth	–	–	SCTGS provide the greatest probability of achieving complete root coverage, increasing KT, better esthetics, and maintaining the root coverage outcomes over time [14].
Alveolar ridge preservation	FGG	2013	RCT	Tooth extraction sites	30	4	Covering the orifice of the extraction socket with FGG could limit the post-operative external contour shrinkage [15].
	FGG	2018	CCT	Tooth extraction sites	22	–	ARP using bovine bone minerals and FGG leads to more scar tissue formation [79].
	SCTG	2019	RCT	Tooth extraction sites	14	12	The application of SCTG partly compensated for the buccal bone loss in alveolar ridge preservation [16].
Eyelid reconstruction	HPMG	2018	Retrospective study	Lower eyelid	34	15	HPMG as supportive material because its structure is similar to the palpebral conjunctiva and has supportive features similar to the tarsal tissue, and also due to its sufficient volume and rapid and invisible wound healing of the donor site [68].
	HPMG	2018	Retrospective study	Lower eyelid	12	53	HPMG combined with the recession of lower eyelid retractors achieves long-term stable outcomes in lower eyelid retraction repair [67].
	HPMG	2017	Retrospective study	Lower eyelid	15	4.5–87.9	An HPMG maintains lower eyelid native epithelial morphology and gives a lasting improvement in most patients [80].
	HPMG	2020	Retrospective study	Lower or upper eyelid	8	12.75	The HPMG provides good and lasting structural support to the eyelid [18].
	HPMG	2021	Retrospective study	Upper eyelid	8	48	The reduction of HPMG thickness helps to achieve a smooth eyelid appearance, and HPMG may stimulate the cornea [17].
	HPMG	2008	Retrospective study	Lower or upper eyelid	107	21	The HPMG for upper and lower eyelid cicatricial entropion surgery provides high symptomatic and anatomical cure rates [81].
Contracted anophthalmic socket reconstruction	HPMG	2019	Retrospective study	Eye socket	4	5–59	The reconstructive technique of using a hard palate-dermis fat composite graft provides excellent volume supplementation and retention of conjunctival fornix configuration in contracted anophthalmic sockets [19].
Nail bed reconstruction	HPMG	2002	Case report	Nail bed	2	7–8	The HPMG without periosteum to a defect of the nail bed contributes to a shorter healing time [20].
	HPMG	2006	Case report	Nail bed	6	1.5–6	Treating nail bed defects with an HPMG is a safe and effective procedure [21].
Lip defect reconstruction	HPMG	2007	Case report	Lip	1	6	HPMG with mucoperiosteal for the reconstruction of the lower lip has the following advantages: less post-operative shrinkage, no residual donor site morbidity, and pleasing aesthetic and functional results [22].
Nasal vestibular stenosis reconstruction	HPMG	1997	Case report	Nasal vestibular	1	–	HPMG is tough, resilient, and easy to harvest. Its ability to resist contractures eliminates the need for post-operative stents, which is particularly useful in the pediatric population [23].
Posttraumatic nasal deformity correction	HPMG	2000	Case series	Nasal	5	7–16	HPMG is applicable in the correction of traumatic nasal deformity, and because of the stiffness of HPMG, it did not collapse in breathing [24].
Hypopharyngeal defect reconstruction	HPMG	1994	Case report	Hypopharynx	1	18	HPMG with mucoperiosteal is a desirable graft material with the following characteristics: its similarity to the surrounding tissue, inherent strength, and minor primary contracture [25].
Tracheal reconstruction	HPMG	2004	Case report	Tracheal	1	12	The inner layer of the trachea was reconstructed with HPMG, and no dirt or sputum was stuck [26].

### 4.1 Applications in dentistry

HPM-derived biomaterials were first used in dentistry for clinical practice. The first proposed FGG and the later SCTG have played an irreplaceable role in various oral soft tissue procedures, including natural teeth or peri-implant KTW augmentation [82], root coverage [83,84], alveolar ridge preservation [15], and increasing vestibulum depth [13].

The keratinized tissue with adequate width and thickness is critical for both natural teeth and dental implants, and receding or absent gingiva affects the function of teeth and severely impacts oral aesthetics [85]. Lack of textured mucosa around implants has been shown to impede oral hygiene, leading to soft tissue inflammation, gingival recession, and attachment loss [86,87]. FGG is the most effective way to restore keratinized gingival width, but due to the frequent color mismatch between FGG and gingiva, its application in the aesthetic area is often limited [13]. With the improvement of periodontal surgical instruments and the advancement of surgical concepts, more and more dentists are using SCTG instead of FGG to obtain better aesthetic results. There is much evidence that SCTG can effectively treat gingival/mucosal recession in natural teeth and implants [14,88], mask discolored roots or visible implant components [10,89], increase soft tissue thickness [12,90], and reconstruct interdental papillae [83,91]. Furthermore, the increasing shift from FGG to SCTG indicates a shift from traditional gingival surgery to periodontal plastic surgery.

The soft tissue after extraction is insufficient to achieve primary wound closure. The exposed wound may interfere with the alveolar ridge preservation and ultimately adversely affect the function and aesthetic results of the implant restoration. In contrast, FGG is the most effective frontal method for site-preserving wound closure. Several studies have shown that vertical and horizontal changes in the alveolar ridge are reduced when FGG is used for extraction wound coverage [15,79,92]. Using SCTG in the aesthetic area avoids the deficiencies associated with the FGG color difference.

The main reason for the widespread use of FGG and SCTG in oral soft tissue surgery is that the HPM has a similar structure to the keratinized gingiva and can be well integrated with the surrounding gingiva. At the same time, the hard palate as a surgical donor area has a simple anatomy with a sufficient thickness of lamina propria. Under local anesthesia, the surgeon can perform the graft extraction without unacceptable complications for the patient.

### 4.2 Applications in eyelid reconstruction

The ideal outcome of eyelid reconstruction surgery should be a removable eyelid, good corneal protection, good aesthetic quality, and acceptable donor-area sequelae. However, complications caused by soft tissue contracture or gravity in lower eyelid reconstruction (e.g., eyelid constriction, ectropion, ptosis) are still common. Selection of the ideal soft tissue graft is a prerequisite to ensure surgical outcomes.

The use of HPMG for eyelid reconstruction was first proposed by Siegel in 1985 [6]. Many studies have demonstrated that HPMG is an attractive autologous option for upper and lower eyelid defects [19,67,93]. HPMG provides the proper flexibility to accommodate the bulging shape of the eye while the epithelial layer of HPMG maintains appropriate rigidity to support the lower eyelid [18]. In addition, HPMG has a wide donor area, insignificant post-operative scarring, and a low complication rate, and the most common complication is donor area bleeding, with an incidence of 2.7–10%. However, it has also been suggested that using HPMG in upper eyelid reconstruction is controversial because it consists of keratinized and stratified squamous epithelium that can irritate the cornea [17].

### 4.3 Applications in other special soft tissue

Compared to the extensive clinical applications in dentistry and eyelid reconstruction, there are relatively few reports of HPM-derived biomaterials in other specific soft tissue areas.

Hatok et al. reported two children with nail bed defects after removing subungual exosomes. After removing the tumor, HPMG without periosteum was transplanted to the nail bed defect area. The patients could enjoy daily life, including exercise, without any symptoms two weeks after surgery, and the nails grew smoothly without complications within 4–5 months [20]. Fernandez-Mejia reported that six patients had seven nails repaired by palatal mucosa grafting, and all repaired nails grew well and improved nail dystrophy [21].

Ito & et al. reported a patient with a lower lip deformity complicated by malignant tumor resection. HPMG with periosteum reconstructed the lower lip well, and the lips closed perfectly without salivary leakage. The reconstructed lower lip had a natural contour with good aesthetic and functional results. The authors noted that this type of graft seems to be a practical option for reconstructing lip deformities. It can be widely used to treat lip deformities caused by tumor removal, trauma, or cleft lip [22].

Maj et al. reported a case of reconstructed nasal vestibule stenosis in an infant using HPMG surgery. Six months after surgery, the nasal vestibule maintained recanalization, nasal growth was expected, and scarring and wound contracture disappeared. Post-operative recurrence was also avoided due to the inherent ability of HPMG to resist constriction and replace the bioprosthesis [23].

Other clinicians have used triangular pectoral flaps and HPMG combined with rib cartilage grafts for tracheal reconstruction, successfully reconstructing the tracheal mucosa, tracheal cartilage, and overlying skin with adequate subcutaneous tissue. In addition, cases of successful treatment of intractable hypopharyngeal scar stenosis using HPMG have been reported in the literature [25].

## 5 Challenges and limitations

In recent decades, clinicians have used autologous soft tissue grafts obtained from the HPM for many types of soft tissue reconstruction. Researchers have also conducted various studies on HPM and other HPM-derived biomaterials, but the full development of HPM-derived biomaterials still needs to overcome various challenges and limitations.

Although the HPM heals faster and has less scarring than skin incisions, it still takes several days to achieve initial healing [94]. HPM-derived biomaterials could be more widely used in clinical practice if the problem of post-operative reaction of donor area wounds could be addressed. No highly effective drugs or topical treatments can further improve donor area wound healing efficiency at this stage.

Previous studies have shown that oral wounds heal faster than the skin, with significantly less inflammation, less hypoxia, faster epithelial formation, finer angiogenic responses, and less scarring [31,32]. Most existing studies have been conducted to compare the differences between oral mucosal and skin wound healing processes. However, for the time being, no suitable factors have been identified from the HPM healing characteristics that can be applied to enhance the healing ability of skin wounds [43].

Several studies have proposed that HPM-derived MSCs have good prospects for application. However, most of the studies at this stage only stop at the isolation, identification, and trilineage differentiation. Few studies have subjected them to bone regeneration and osseointegration in animal models [9]. Long-term studies are still needed to elucidate the harvesting efficiency of HPM-derived MSCs and the yield and potency of each tissue before clinical application. In addition, researchers need to conduct comprehensive work to confirm its safety and expand its application.

The development of HPM-derived biomaterials is still in the early stages, and the development of new materials requires the combination of the properties of the HPM itself with the new technologies available. However, there are still limitations in detecting the components and properties of HPM. In addition, no studies have reported the biocompatibility and immunogenicity associated with HPM-derived biomaterials.

## 6 Future directions

Soft tissue grafts of HPM origin have been widely used in clinical practice with irreplaceable results. However, the overall research on other HPM-derived biomaterials is still preliminary. This section highlights the challenges and prospects in the field of HPM-derived biomaterials (Table 2).

**Table 2:** Future targets and possible research directions of HPM-derived biomaterials.

Future targets	Possible research directions	Specific examples of existing or forthcoming solutions
Enhance the efficiency of wound healing	Clinical minimally invasive techniques	Trap-door technique [65]
		Single-incision technique [66]
	Application of drugs	Phenytoin [95]
		Medicinal plant extract [94]
		Lectin Artin M [96]
	Physiotherapeutic	Low-intensity electrotherapy [97]
Research and applications of wound healing mechanisms	Rapid healing ability	Accelerate skin wound healing
	Scarless healing	Skin scarring removal
Research on the applications of MSCs	Tissue regeneration	Bone regeneration
		Implant osteosynthesis
		Periodontal tissue regeneration
	Tissue homeostasis	Inflammatory regulation
	Neuron repair	Repair of brain tissue damage
Develop new materials	Decellularized biomaterials	Decellularized HPMG
		Bioink material
	Genetically modified biomaterials	Transgenic stem cells
		Transgenic decellularized materials

### 6.1 Enhance the efficiency of wound healing

Clinically, the main methods to improve the efficiency of wound healing include minimally invasive surgery, topical application of drugs, and local physical therapy [98,99]. Researchers have also conducted studies to promote to healing efficiency of the HPM.

A more minimally invasive surgical approach is undoubtedly a more direct and effective way to improve donor wound healing efficiency. Edel et al. proposed the trap-door technique, which preserves the epithelial layer of the palate while effectively obtaining SCTG to facilitate one-stage donor wound healing and thus reduce post-operative discomfort [65]. In 1999, Hürzeler et al. proposed the single-incision technique, which reduces the vertical incision, preserves the blood supply of the superficial flap compared with the trapdoor technique, and provides better one-stage healing [66]. However, both methods require adequate HPM thickness compared to the conventional method of FGG excision through epithelial debridement to obtain SCTG. Some clinical studies have found that the thicker the palatal mucosa, the thicker the post-operative residual mucosa, the faster the wound healing, and the less post-operative reaction [100,101].

Applying medication to the wound is also an effective way to enhance healing efficiency. Another clinical trial showed that topical application of phenytoin to palatal wounds improved the healing outcome and reduced post-operative adverse effects [95]. Another study also suggested that topical medicinal plant extracts (MPE) to wounds in the mucosal donor area of the patient's hard palate could promote hemostasis and early wound healing and reduce patient pain [94]. Another animal study found that after applying Artin-M gel to HPM wounds in dogs, Artin-M recruited neutrophils and promoted cell proliferation, thereby promoting wound healing in palatal mucosal injuries [96].

Skin or mucosal wounds resemble a natural endogenous battery that generates an electric current, and electrical stimulation can mimic this current at the wound and initiate or accelerate wound repair. One study using electrical stimulation in mucosal wounds of the hard palate in mice found that electrical stimulation accelerated the effect of early wound closure and positively affected the inflammatory markers IL-6, interleukin-10 (IL-10), tumor necrosis factor-α (TNF-α), and VEGF [102]. A recent clinical randomized controlled trial also showed that using a low-intensity electrotherapy protocol can accelerate wound healing in the palate and reduce patient discomfort after FGG surgery [97].

Improving the efficiency of wound healing in the donor area and reducing complications is essential for the clinical applications of HPM-derived biomaterials. Possible improvements include optimizing clinical techniques, developing related drugs, and local physiotherapy.

### 6.2 Research and applications of wound healing mechanisms

To identify and explain the mechanisms that accelerate oral wound healing, several researchers have analyzed changes in gene expression during oral mucosal and skin wound resolution in healthy individuals. They identified factors that SOX2 and PITX1 regulate networks involved in wound closure and reprogramed skin keratinocytes through SOX2 and PITX1 to make these cells present the characteristics of oral keratinocytes, thus increasing cell migration and improving wound healing *in vivo* [31].

Although more studies are needed to identify the specific pathways in HPM wound healing and their potential for wound repair and tissue regeneration, the molecular basis and signaling pathways of HPM wound healing may be considered for therapeutic application to non-oral mucosal sites, such as accelerated skin wound healing, reduction of scar formation, and recalcitrant scar treatment.

### 6.3 Research on the applications of MSCs

Stem cell-based therapies are an essential branch of regenerative medicine whose ultimate goal is to enhance the repair mechanisms by stimulating, modulating, and regulating endogenous stem cell populations and replenishing cell pools to achieve tissue homeostasis and regeneration [103]. It is well documented that MSCs have a solid immunomodulatory effect and migration capacity and play a vital function in regulating immune responses and the development of multiple diseases [104]. The safety and efficacy of MSCs therapies have recently been demonstrated in clinical trials [105].

Existing studies have extracted MSCs from the hard palate periosteum [8], subepithelial adipose tissue [59], and lamina propria [9], respectively. It has been suggested that MSCs of hard palate adipose origin may have better osteogenic capacity than subcutaneous fat [106]. A recent study prepared cellular polymers based on PMSCs and demonstrated through animal experiments that the polymers significantly promoted bone regeneration and implant osseointegration [9]. It was also suggested that MSCs derived from HPM could increase the local regenerative potential of periodontal tissues. Moreover, PMSCs possess a stronger anti-inflammatory capacity than other sources of MSCs, suggesting that their corresponding exosomes could regulate inflammation [9,107]. After being cultured in a neuronal differentiation medium, the HPM-derived MSCs gradually transformed into neuronal morphology. They expressed multiple neuronal markers, suggesting that the MSCs may have neuronal repair ability and applications in brain tissue injury repair [76].

HPM-derived MSCs are relatively minimally invasive and ideal for stem cell sources. Based on the characteristics of existing PMSCs, the fields of tissue regeneration, tissue homeostasis, and neuronal repair may be the future research directions.

Moreover, extracellular vesicles from MSCs can modulate inflammatory responses, immune regulation, angiogenesis, coagulation, extracellular matrix remodeling, and apoptosis [106,107]. Obtaining extracellular vesicles with specific properties may be possible from HPM-derived MSCs.

### 6.4 Development of new materials

The HPM consists of the epithelial layer, lamina propria, and submucosa, of which the lamina propria is the thickest and consists mainly of dense connective tissue composed mainly of a large number of collagen fibers and a small number of elastic fibers [55,56]. In addition to the expansion of applications of MSCs, new materials can be developed based on the composition of the HPM in combination with new technologies.

In recent years decellularized materials have played an essential role in tissue repair [108]. Decellularized extracellular matrix (dECM) can retain the physicochemical properties or complex biological and physical characteristics of native ECM [109]. Decellularization aims to remove as many cells as possible and minimize their immunogenicity and cytotoxicity, usually using chemical, physical and biological methods [110]. Due to continuous advances in research and technological innovations, several decellularized biomaterials have been used in commercial applications and clinical treatments with satisfactory therapeutic results [111].

The HPM donor area is often only adequate for tiny autologous grafts. The decellularization technique can preserve the physical characteristics of native ECM and associated cytokines [112]. Decellularized HPMG may make the development of allogeneic or xenogeneic HPM-derived materials feasible and create the conditions for the clinical application of HPM in oral and maxillofacial tissue regeneration, skin and wound healing applications, cartilage and bone tissue engineering, and neural tissue regeneration.

Furthermore, dECM has been identified as a promising material for three-dimensional (3D) bioprinting applications since dECM offers excellent cellular activity because it retains ECM components [113]. 3D bioprinting using dECM-based bioink can revolutionize the engineering of physiologically relevant tissues and organs [114]. If decellularized HPMG can preserve the physical characteristics of native HPMG and related cytokines, and it may become a promising bioink material.

Genetic modification, overexpression or knockout of specific genes via different vectors and vehicles, not only alters the properties of the biomaterials themselves but also participates in the interactions between the biomaterial and the seeded cells [115]. Genetic modification may allow for more personalized HPM-derived materials, including transgenic stem cells and tissue graft materials. Furthermore, combining decellularization and genetic modifications may offer HPM more advantages in biomedical research and relevant applications.

## 7 Conclusion

In recent years, the HPM has been known as an ideal donor area for autografts because of its high wound-healing ability, low clinical difficulty, and large donor area. Researchers have obtained soft tissue grafts such as FGG, SCTG, and HPMG from HPM with exceptional mechanical properties, which have played an important role in dental, eyelid repair, and other special soft tissue defect repair. In addition, various MSCs and derivatives extracted from HPM are in the research stage. Future research will focus on enhancing the efficiency of wound healing, further elucidating wound healing mechanisms, applying MSCs, and developing new materials derived from HPM. The HPM, as a donor area for small-scale autografts, heals quickly, has less scarring, can be repeatedly extracted, and is expected to become an essential source of human autologous biomaterials (tissues and cells) after a deeper investigation.

## Declaration of competing interest

The authors declare that they have no known competing financial interests or personal relationships that could have appeared to influence the work reported in this paper

## Data Availability

No data was used for the research described in the article.
